# Cocktail δ-integration: a novel method to construct cellulolytic enzyme expression ratio-optimized yeast strains

**DOI:** 10.1186/1475-2859-9-32

**Published:** 2010-05-14

**Authors:** Ryosuke Yamada, Naho Taniguchi, Tsutomu Tanaka, Chiaki Ogino, Hideki Fukuda, Akihiko Kondo

**Affiliations:** 1 Department of Chemical Science and Engineering, Graduate School of Engineering, Kobe University, 1-1 Rokkodaicho, Nada-ku, Kobe, Hyogo 657-8501, Japan; 2 Organization of Advanced Science and Technology, Kobe University, 1-1 Rokkodaicho, Nada-ku, Kobe, Hyogo 657-8501, Japan

## Abstract

**Background:**

The filamentous fungus *T. reesei *effectively degrades cellulose and is known to produce various cellulolytic enzymes such as β-glucosidase, endoglucanase, and cellobiohydrolase. The expression levels of each cellulase are controlled simultaneously, and their ratios and synergetic effects are important for effective cellulose degradation. However, in recombinant *Saccharomyces cerevisiae*, it is difficult to simultaneously control many different enzymes. To construct engineered yeast with efficient cellulose degradation, we developed a simple method to optimize cellulase expression levels, named cocktail δ-integration.

**Results:**

In cocktail δ-integration, several kinds of cellulase expression cassettes are integrated into yeast chromosomes simultaneously in one step, and strains with high cellulolytic activity (i.e., expressing an optimum ratio of cellulases) are easily obtained. Although the total integrated gene copy numbers of cocktail δ-integrant strain was about half that of a conventional δ-integrant strain, the phosphoric acid swollen cellulose (PASC) degradation activity (64.9 mU/g-wet cell) was higher than that of a conventional strain (57.6 mU/g-wet cell). This suggests that optimization of the cellulase expression ratio improves PASC degradation activity more so than overexpression.

**Conclusions:**

To our knowledge, this is the first report on the expression of cellulase genes by δ-integration and optimization of various foreign genes by δ-integration in yeast. This method should be very effective and easily applied for other multi-enzymatic systems using recombinant yeast.

## Background

Given the eventual exhaustion of fossil fuels and environmental issues such as global warming and acid rain, utilization of biomass as a source of fuels and fine chemicals has recently become an attractive option. Utilization of biomass, especially cellulosic materials, is desirable because it is abundant, inexpensive, renewable, and has favorable environmental properties. Therefore, an efficient and cost-effective method for degradation of cellulosic materials into glucose to produce alternative fuels or other fine chemicals is required.

Efficient degradation of cellulose requires a synergistic reaction of the cellulolytic enzymes endoglucanase (EG), cellobiohydrolase (CBH), and β-glucosidase (BGL). Although there are many reports concerning cost reduction for cellulosic material degradation using recombinant bacteria, fungi, and yeast [1-3], cellulase degradation efficiency has not been improved enough. We previously reported direct ethanol production from phosphoric acid swollen cellulose (PASC) using a yeast strain co-displaying *Trichoderma reesei *EGII, CBHII, and *Aspergillus aculeatus *BGL1 [4]. This method however requires higher cellulase degradation for cost-effective ethanol production from cellulosic materials.

The filamentous fungus *T. reesei *degrades cellulose effectively and is known to produce various types of cellulolytic enzymes and control their expression levels simultaneously depending on the environment; the ratio of the cellulases and their synergetic effects are important for effective cellulose degradation [5,6]. Promoter engineering is one method to control cellulase expression levels [7-9]. However, the variety of promoters adequate for cellulase overexpression is limited, and expression levels by each promoter vary with environmental conditions such as glucose concentration or the phase of cell growth [7]. Furthermore, the optimum expression ratio of various cellulolytic enzymes for efficient cellulose degradation is unknown, and the ratio will differ depending on the content of the cellulosic material.

In this study, to construct engineered yeast with efficient cellulose degradation, we developed a simple method to optimize cellulase expression levels, called cocktail δ-integration. The δ-integration method is known as multicopy-integration in yeast [10]. In cocktail δ-integration, several kinds of cellulase expression cassettes are integrated into yeast chromosomes simultaneously in one step, and strains with high cellulolytic activity (i.e., expressing the optimum ratio of cellulases) can be easily obtained. The goal of this study was to create a cellulase expression-optimized yeast strain for efficient degradation of cellulose using our novel cocktail δ-integration method.

## Materials and methods

### Strains, plasmids, and media

Table 1 summarizes the genetic properties of all strains used in this study. In brief, the host for recombinant DNA manipulation was the *Escherichia coli *strain NovaBlue (Novagen, Madison, WI, USA), and cellulolytic enzymes were expressed in the haploid yeast strain *S. cerevisiae *MT8-1 [11].

**Table 1:** Characteristics of bacterial and yeast strains used in this study

Strain or plasmid	Relevant features	Reference
Bacterial strain		
*E. coli *Novablue	*endA1 hsdR17(r*_*K12 *_^-^*m*_*K12 *_^+^) *supE44 thi-I gyrA96 relA1 lac recA1/F *[*proAB *^+ ^*lacI *^q ^ZΔM15::Tn*10*(Tet^r^)]	Novagen
*S. cerevisiae *yeast strains		
MT8-1	*ade his3 leu2 trp1 ura3*	[11]
MT8-1/IBEC	*ade leu2 *Integration of β-glucosidase, endoglucanase, and cellobiohydrolase gene	this study
MT8-1/δBEC	*ade leu2 *δ-Integration of β-glucosidase, endoglucanase, and cellobiohydrolase gene	this study
MT8-1/cocδBEC1	*ade his3 leu2 ura3 *cocktail δ-Integration of β-glucosidase, endoglucanase, and cellobiohydrolase gene	this study
MT8-1/cocδBEC2	*ade his3 leu2 *cocktail δ-Integration of β-glucosidase, endoglucanase, and cellobiohydrolase gene	this study
MT8-1/cocδBEC3	*ade leu2 *cocktail δ-Integration of β-glucosidase, endoglucanase, and cellobiohydrolase gene	this study

*E. coli *transformants were grown in Luria-Bertani medium (10 g/l tryptone, 5 g/l yeast extract, and 5 g/l NaCl) supplemented with 100 μg/ml ampicillin. Yeast transformants were screened in SD medium (6.7 g/l yeast nitrogen base without amino acids [Difco Laboratories, Detroit, MI, USA] and 20 g/l glucose) supplemented with the appropriate amino acids and nucleic acids. For cocktail δ-integration, SPASC medium (6.7 g/l of yeast nitrogen base without amino acids [Difco Laboratories] and 10 g/l PASC) supplemented with the appropriate amino acids and nucleic acids was used. Yeast cells were aerobically cultured in SD or YPD medium (10 g/l yeast extract, 20 g/l Bacto-peptone [Difco Laboratories], and 20 g/l glucose).

### Plasmid construction

**Figure 1:**
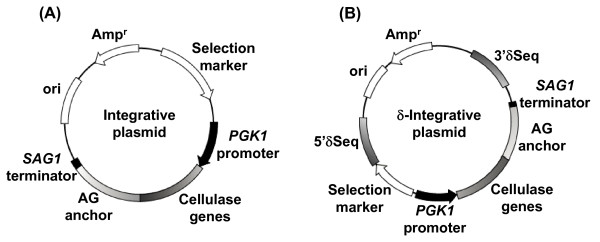
**Genetic property of cellulase expressing plasmids**. (A) Plasmid for integration. (B) Plasmid for δ-integration.

Table 2 and Figure 1 represent the genetic properties of all plasmids used in this study. The integrative plasmid pIHAGBGL-NotI [12] was used for surface expression of BGL1.

**Table 2:** Characteristics of plasmids used in this study

Plasmid	Recombination type	Selection marker	Expressing cellulase gene
pIHAGBGL-NotI	Integration	*HIS3*	β-Glucosidase
pIU-PGAGEG		*URA3*	Endoglucanase
pIW-PGAGCBH		*TRP1*	Cellobiohydrolase
pδW-PGAGBGL	δ-Integration	*TRP1*	β-Glucosidase
pδU-PGAGBGL		*URA3*	β-Glucosidase
pδH-PGAGBGL		*HIS3*	β-Glucosidase
pδW-PGAGEG		*TRP1*	Endoglucanase
pδU-PGAGEG		*URA3*	Endoglucanase
pδH-PGAGEG		*HIS3*	Endoglucanase
pδW-PGAGCBH		*TRP1*	Cellobiohydrolase
pδU-PGAGCBH		*URA3*	Cellobiohydrolase
pδH-PGAGCBH		*HIS3*	Cellobiohydrolase

The integrative plasmid for surface expression of *T. reesei *EGII was constructed as follows: The NotI-NotI DNA fragment encoding the *S. cerevisiae PGK1 *promoter, a secretion signal sequence, the *T. reesei *endoglucanase gene, the 3'-half of the α-agglutinin gene, and the PGK1 terminator was amplified from pIWAGEGII [12] by PCR with the primers pPGKF(NotI): 5'-ATGCATGCGGCCGCCGATTTGGGCGCGAATCCTT-3' and tAGR(NotI): 5'-ATAAGAATGCGGCCGCTTTGATTATGTTCTTTCTATTTGAATGAGATATG-3'. The fragment was digested with NotI and inserted into the NotI site of the plasmid pRS406 (Stratagene). The resultant plasmid was named pIU-PGAGEG.

The integrative plasmid for surface expression of *T. reesei *CBHII was constructed as follows: The NotI-NotI DNA fragment encoding the *S. cerevisiae PGK1 *promoter, a secretion signal sequence, the *T. reesei *cellobiohydrolase gene, the 3'-half of the α-agglutinin gene, and the PGK1 terminator was amplified from pIHAGCBHII [12] by PCR with the primers pPGKF(NotI) and tAGR(NotI). The fragment was digested with NotI and inserted into the NotI site of the plasmid pRS404 (Stratagene). The resultant plasmid was named pIW-PGAGCBH.

The δ-integrative basic plasmid pδH (*HIS3 *as a selective marker) was constructed as follows: The XhoI-XhoI DNA fragment encoding the large portion of the promoter-deficient *HIS3 *(*HIS3d*) marker gene was amplified from pRS403 (Stratagene) by PCR using the primers *HIS3*dF (XhoI): 5'-ACCGTCGACCTCGAGCTTCGAAGAATATACTAAAA-3' and *HIS3*dR (XhoI): 5'-GGGCCCCCCCTCGAGTCGAGTTCAAGAGAAAAAAA-3'. The fragment was inserted into the XhoI site of plasmid pδseq [13] and the resulting plasmid was named pδH.

The δ-integrative plasmid for surface expression of BGL1 was constructed as follows: The NotI-NotI DNA fragment encoding the *S. cerevisiae PGK1 *promoter, a secretion signal sequence, the *A. aculeatus *β-glucosidase gene, the 3'-half of the α-agglutinin gene, and the PGK1 terminator was obtained by NotI digestion of pIHAGBGL-NotI and inserted into the NotI sites of the plasmids pδU, pδW [13], and pδH, respectively. The resultant plasmids were named pδU-PGAGBGL, pδW-PGAGBGL, and pδH-PGAGBGL, respectively.

The δ-integrative plasmids for surface expression of EGII were constructed as follows: The NotI-NotI DNA fragment encoding the *S. cerevisiae PGK1 *promoter, the *T. reesei *endoglucanase gene with a secretion signal sequence, and the 3'-half of the α-agglutinin gene with a terminator was obtained by NotI digestion of pIU-PGAGEG and inserted into the NotI sites of the plasmids pδU, pδW, and pδH to generate pδU-PGAGEG, pδW-PGAGEG, and pδH-PGAGEG, respectively.

The δ-integrative plasmids for surface expression of CBHII were constructed as follows: The NotI-NotI DNA fragment encoding the *S. cerevisiae PGK1 *promoter, the *T. reesei *cellobiohydrolase gene with a secretion signal sequence, and the 3'-half of the α-agglutinin gene with a terminator was obtained by NotI digestion of pIW-PGAGCBH and inserted into the NotI sites of the plasmids pδU, pδW, and pδH to generate pδU-PGAGCBH, pδW-PGAGCBH, and pδH-PGAGCBH, respectively.

### Yeast transformation and cocktail δ-integration

In conventional integration and δ-integration, plasmids were transformed into *S. cerevisiae *MT8-1 using lithium acetate as described [14]. The transformants with the highest cellulolytic activity were selected from several colonies and used in subsequent experiments.

In cocktail δ-integration, identical amounts of three co-marked δ-integrative plasmids (over 20 μg of each plasmid), pδW-PGAGBGL, pδW-PGAGEG, and pδW-PGAGCBH, were mixed and transformed simultaneously. The transformants were spread on SPASC medium, and the transformant with the highest cellulolytic activity was selected from tested over 100 numbers of large colonies. The selected transformant was named MT8-1/cocδBEC1 and used in the subsequent transformation, named repeated cocktail δ-integration. Similar to the first step, identical amounts of three co-marked δ-integrative plasmids, pδU-PGAGBGL, pδU-PGAGEG, and pδU-PGAGCBH, were mixed and transformed into MT8-1/cocδBEC1 simultaneously. The selected transformant with the highest cellulolytic activity on SPASC medium was named MT8-1/cocδBEC2. Finally, identical amounts of three co-marked δ-integrative plasmids, pδH-PGAGBGL, pδH-PGAGEG, and pδH-PGAGCBH, were mixed and transformed into MT8-1/cocδBEC2 simultaneously. The selected transformant with the highest cellulolytic activity on SPASC medium was named MT8-1/cocδBEC3.

### Enzyme assay

Yeast cells cultivated in YPD medium for 72 h at 30°C and collected by centrifugation at 3,000 × *g *for 5 min at 4°C and washed with distilled water twice were used for determination of β-glucosidase and PASC degradation activity.

β-Glucosidase activity was measured in 50 mM sodium acetate buffer (pH 5.0) at 30°C with 2 mM p-nitrophenyl-β-D-glucopyranoside (Nacalai Tesque, Inc., Kyoto, Japan) as the substrate. The wet cell concentration of the reaction mixture was adjusted to 1 g-wet cell/l. After the reaction, supernatants were separated by centrifugation at 10 000 × *g *at 4°C. The amount of released p-nitrophenol was determined by measuring the absorbance at 400 nm. One unit of β-glucosidase activity was defined as the amount of enzyme producing 1 μmol/min p-nitrophenol at 30°C, pH 5.0.

PASC degradation activity was determined by hydrolysis of 1 g/l amorphous cellulose in 50 mM sodium acetate buffer (pH 5.0) at 50°C. PASC was prepared from Avicel PH-101 (Fluka Chemie GmbH, Buchs, Switzerland) as amorphous cellulose [15]. The wet cell concentration of the reaction mixture was adjusted to 50 g-wet cell/l. After hydrolysis, the supernatant was separated by centrifugation for 5 min at 10 000 × *g *at 4°C, and the produced glucose concentration was measured using the Glucose CII test Wako (Wako Pure Chemical, Osaka). One unit of PASC degradation activity was defined as the amount of enzyme producing 1 μmol/min glucose at 50°C, pH 5.0.

### Quantification of integrated copy numbers by real-time PCR

The integrated copy number of each recombinant strain was quantified using real-time PCR. Template genome DNA was isolated from yeast cells cultivated in SD medium for 72 h at 30°C using a YeaStar Genomic DNA kit (Zymo Research, Orange, CA). The 3 sets of PCR primers, BGL 761F: 5'-CTTCCAGGGCTTTGTGATGTC-3' and BGL 858R: 5'-AGGTGATATCGCCAGGCATT-3', and EGII 694F: 5'-CCACGGTCCAAGAGGTTGTAA-3' and EGII 774R: 5'-GCCAATCATTTCCAGGCAAA-3', and CBHII 571F: 5'-GGCGTCGCCAAATATAAGAACT-3' and CBHII 653R: 5'-ATAACCAGGAGGGTCCGGATA-3' were used to detect the BGL, EG, and CBH genes respectively. Quantitative real-time PCR was performed using an ABI PRISM 7000 Sequence Detection System (Applied Biosystems, Foster City, CA) with Thunderbird SYBR qPCR Mix (Toyobo, Osaka, Japan). The normalized gene copy number was calculated by the standard curve method with the *PGK1 *gene as the house keeping gene.

## Results

### General strategy of repeated cocktail δ-integration

Cocktail δ-integration works as follows. First, three cellulase genes, BGL, EG, and CBH, were introduced into yeast genomes simultaneously by using the δ-integration method with one marker gene. As a result, a pool of recombinants with various genes having a different number of copies was constructed. Then a transformant with optimized cellulase expression was selected from the recombinant pool by its ability to form colonies on SPASC medium and its cellulolytic activity. Using the selected transformant, a 2nd round of cocktail δ-integration was carried out using a different marker gene to obtain a transformant with higher PASC degradation ability. After a 3rd round of cocktail δ-integration, the resulting transformant had almost the same PASC degradation activity as the 2nd round transformant, showing that PASC degradation activity was saturated (see the following section). This strategy is referred to as repeated cocktail δ-integration.

### Construction of yeast strains

**Figure 2:**
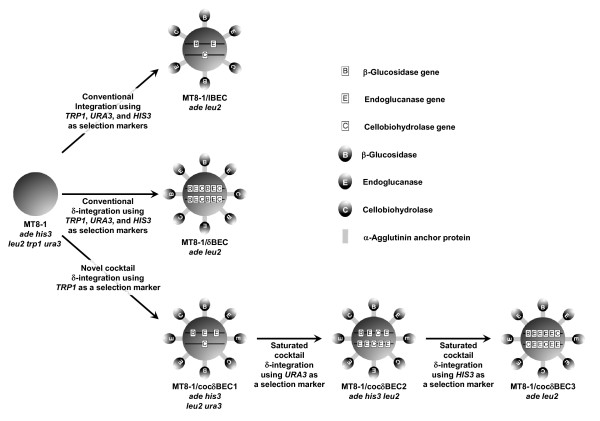
**Strategy for constructing cellulase expressing yeast strains**.

The five recombinant yeast strains constructed in this study are shown in Figure 2. The yeast strain MT8-1/IBEC has BGL, EG, and CBH genes integrated into its chromosome using a conventional integration method. The δ-integrated strain MT8-1/δBEC and cocktail δ-integrated strains MT8-1/cocδBEC1, MT8-1/cocδBEC2, and MT8-1/cocδBEC3 have the same genes integrated using the δ-integration and cocktail δ-integration method, respectively.

### β-Glucosidase and PASC degradation activities

**Figure 3:**
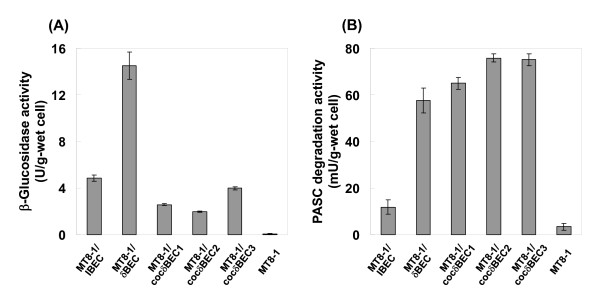
**(A) β-Glucosidase and (B) PASC degradation activities of cellulase expressing yeast strains**. MT8-1/IBEC, conventional integrated strain; MT8-1/δBEC, conventional δ-integrated strain; MT8-1/cocδBEC1, cocktail δ-integrated strain; MT8-1/cocδBEC2 and MT8-1/cocδBEC3, repeated cocktail δ-integrated strain; MT8-1, wild-type strain. Data are averages from five independent experiments.

To investigate the effect of cocktail δ-integration, the BGL and PASC degradation activities of each recombinant yeast cell were measured after cultivation on YPD medium. As shown in Figure 3A, the BGL activity of the conventional δ-integrated strain MT8-1/δBEC (14.5 U/g-wet cell) was 3-fold higher than that of the conventional integrated strain MT8-1/IBEC (4.9 U/g-wet cell). The BGL activities of all cocktail δ-integrated strains (MT8-1/cocδBEC1, MT8-1/cocδBEC2, and MT8-1/cocδBEC3) were lower than that of the conventional integrated strain MT8-1/IBEC.

As shown in Figure 3B, the PASC degradation activity of the conventional δ-integrated strain MT8-1/δBEC (57.6 mU/g-wet cell) was 5-fold higher than that of the conventional integrated strain MT8-1/IBEC (11.8 U/g-wet cell). Alternatively, the PASC degradation activity of the cocktail δ-integrated strain MT8-1/cocδBEC1 was approximately 64.9 mU/g-wet cell, which was higher than that of MT8-1/δBEC. The PASC degradation activities of repeated cocktail δ-integrated strains MT8-1/cocδBEC2 and MT8-1/cocδBEC3 were respectively 75.8 and 75.1 mU/g-wet cell, which are almost the same as each other and significantly improved compared to MT8-1/δBEC.

### Integrated copy numbers of cellulolytic genes

**Figure 4:**
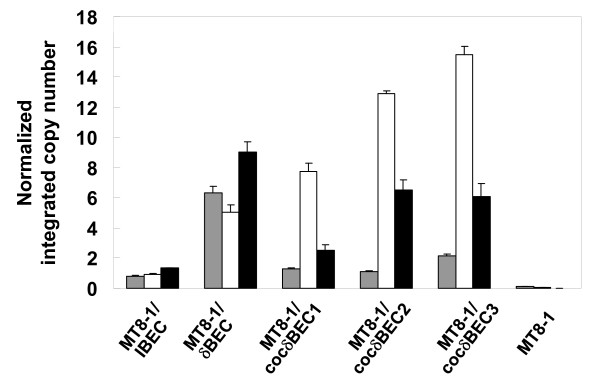
**Determination of cellulase gene copy number of cellulase-expressing yeast strains**. Gray bar, β-glucosidase; white bar, endoglucanase; black bar, cellobiohydrolase. MT8-1/IBEC, conventional integrated strain; MT8-1/δBEC, conventional δ-integrated strain; MT8-1/cocδBEC1, cocktail δ-integrated strain; MT8-1/cocδBEC2 and MT8-1/cocδBEC3, repeated cocktail δ-integrated strain; MT8-1, wild-type strain. Data are averages from three independent experiments.

To investigate the integrated copy number of transformants, real-time PCR was conducted using each transformant genomic DNA as the template. Figure 4 shows the copy number of cellulase integrated strains. As expected, the number of each integrated gene (BGL, EG, and CBH) in the conventional integrated strain MT8-1/IBEC was estimated to be 1. On the other hand, the number of integrated BGL, EG, and CBH genes in the conventional δ-integrated strain MT8-1/δBEC was estimated to be 6, 5, and 9, respectively. The integrated number of BGL, EG, and CBH genes in the cocktail δ-integrated strain MT8-1/cocδBEC1 was estimated to be 1, 8, and 2, respectively, and the EG copy number was increased preferentially after the 2nd and 3rd rounds of cocktail δ-integration.

## Discussion

We developed a high performance cellulolytic yeast strain via a novel δ-integration method by optimizing the expression ratio of three types of cellulase genes. To our knowledge, this is the first report concerning the expression of cellulase genes by δ-integration and optimization of various foreign genes by δ-integration in yeast.

Table 3 shows a summary of results in this study. The BGL activity of the conventional δ-integrated strain MT8-1/δBEC was the highest among all of the strains constructed in this study. Although all three cocktail δ-integrated strains (MT8-1/cocδBEC1, MT8-1/cocδBEC2, and MT8-1/cocδBEC3) have lower BGL activity, they show higher PASC degradation activity compared to MT8-1/δBEC. In addition, nearly all the produced soluble sugar from PASC in all strains was glucose, which was confirmed by the Somogyi-Nelson method [16] (data not shown). This clearly shows that BGL activity is sufficient in all strains constructed in this study; however, the EG and CBH activities are insufficient for efficient PASC degradation. These results correspond to the low copy number of BGL in optimized MT8-1/cocδBEC1, MT8-1/cocδBEC2, and MT8-1/cocδBEC3 constructed using repeated cocktail δ-integration (Figure 4).

**Table 3:** Summary of results

Strain	BGL activity (U/g-wet cell)	PASC degradation activity (mU/g-wet cell)	Expected copy number of BGL	Expected copy number of EG	Expected copy number of CBH	Toal integrated copy number	Recombination number
MT8-1/IBEC	5	12	1	1	1	3	3
MT8-1/δBEC	15	58	6	5	9	20	3
MT8-1/cocδBEC1	3	65	1	8	2	11	1
MT8-1/cocδBEC2	2	76	1	13	6	20	2
MT8-1/cocδBEC3	4	75	2	16	6	24	3
MT8-1	0	3	0	0	0	0	0

Although the total integrated gene copy number of MT8-1/cocδBEC1 was about half that of MT8-1/δBEC, the PASC degradation activity was higher (Table 3). This suggests that optimization of the cellulase expression ratio improves PASC degradation more so than overexpression. Additionally, after the 2nd and 3rd rounds of cocktail δ-integration, the integrated gene copy numbers as well as PASC degradation activity reached a plateau. These results show that uses of repeated cocktail δ-integration can optimize the cellulase expression ratio.

The copy number of integrated EG in the cocktail δ-integrated yeast, MT8-1/cocδBEC3, was the highest compared to that of CBH or BGL (Table 3). This result indicates the importance of EG expression for efficient PASC degradation. Many reports have suggested that amorphous cellulose such as PASC and β-glucan can be degraded into glucose by EG and BGL activity without CBH [15,17]. CBH activity is more important than EG activity for efficient degradation of crystalline cellulose such as Avicel, and EG activity is more important in the degradation of amorphous cellulose such as PASC [18]. The fact that the integrated EG copy number was increased preferably for PASC degradation is consistent with these previous reports [15,17,18].

One advantage of our cocktail δ-integration method is that optimization of cellulase expression levels for efficient cellulose degradation can be achieved without knowing the optimum cellulase expression ratio. Although the filamentous fungus *T. reesei*, which effectively degrades cellulose, simultaneously controls the expression levels of various cellulolytic enzymes [19], the expression levels and activities of various individual enzymes are still unknown. In addition, the optimum expression ratio varies depending on the cellulosic material and degradation conditions. For the novel cocktail δ-integration method developed in this study, in which we only prepare δ-integrative vectors for a target substrate such as PASC, we can construct a target protein expressing strain with an optimum ratio under the desired conditions. Additionally, using this cocktail δ-integration method, several genes are introduced simultaneously with only a single recombination operation, and the strain with the highest activity can be improved by repeated cocktail δ-integration. This simple procedure not only reduces time and effort, but also facilitates the construction of recombinant industrial yeast strains because of their weak recombinant host character such as lacking of auxotrophic marker [20].

The conventional integration method is not adequate for the optimization of expression because of the low integrated copy number and expression level (Figures 3 and 4). Although conventional δ-integration or 2 μ-based multicopy plasmid type recombination allow for overexpression of target genes, it is difficult to control and optimize the expression levels of each gene with these methods. Our cocktail δ-integration method has advantage in that it optimizes the ratio of cellulase expression levels with high cellulolytic activity.

## Conclusions

We constructed three strains of cellulase gene expression-optimized yeast via a novel cocktail δ-integration method. This method should be very effective and easily applied to other multi-enzymatic systems like the degradation of hemicellulose [21]. In addition, it can also be easily applied to the construction of recombinant strains using industrial yeast because several genes are integrated simultaneously in one step.

## Abbreviations

BGL: β-glucosidase; EG: endoglucanase; CBH: cellobiohydrolase; PASC: Phosphoric acid swollen cellulose; PCR: polymerase chain reaction

## Competing interests

The authors declare that they have no competing interests.

## Authors' contributions

R.Y. designed and performed the experiments. N.T. performed the experiments. R.Y. and T.T. wrote the paper. C.O., H.F., A.K. commented and supervised on the manuscript. All the authors approved the final manuscript.
